# Safe-Site Effects on Rhizosphere Bacterial Communities in a High-Altitude Alpine Environment

**DOI:** 10.1155/2014/480170

**Published:** 2014-06-04

**Authors:** Sonia Ciccazzo, Alfonso Esposito, Eleonora Rolli, Stefan Zerbe, Daniele Daffonchio, Lorenzo Brusetti

**Affiliations:** ^1^Department of Food, Environmental and Nutritional Sciences (DeFENS), University of Milan, Via Celoria 2, 20133 Milan, Italy; ^2^Faculty of Science and Technology, Free University of Bozen-Bolzano, Piazza Università 5, 39100 Bolzano, Italy

## Abstract

The rhizosphere effect on bacterial communities associated with three floristic communities (RW, FI, and M sites) which differed for the developmental stages was studied in a high-altitude alpine ecosystem. RW site was an early developmental stage, FI was an intermediate stage, M was a later more matured stage. The N and C contents in the soils confirmed a different developmental stage with a kind of gradient from the unvegetated bare soil (BS) site through RW, FI up to M site. The floristic communities were composed of 21 pioneer plants belonging to 14 species. Automated ribosomal intergenic spacer analysis showed different bacterial genetic structures per each floristic consortium which differed also from the BS site. When plants of the same species occurred within the same site, almost all their bacterial communities clustered together exhibiting a plant species effect. Unifrac significance value (*P* < 0.05) on 16S rRNA gene diversity revealed significant differences (*P* < 0.05) between BS site and the vegetated sites with a weak similarity to the RW site. The intermediate plant colonization stage FI did not differ significantly from the RW and the M vegetated sites. These results pointed out the effect of different floristic communities rhizospheres on their soil bacterial communities.

## 1. Introduction

A glacier foreland after glacier retreat can be considered a cold desert, being composed of habitats characterized by severe climatic regimes and barren substrate with low total carbon and nitrogen contents [1]. Rock cracks, concave surfaces, and little depressions could ensure protection from wind, cold, and other harsh environmental conditions [2, 3] helping the accumulation of nutrients and the growth of pioneer plants. Safe-sites are defined as little areas, often surrounded by big stones, filled up of stone debris or mineral mud [4]. Here, opportunistic pioneer plants could settle down and form first floristic consortia, significantly affected by the geochemistry of the lytic material. Indeed, physical and biogeochemical weathering processes provide soils of soluble nutrients and when the plant colonization on parent materials occurs, the development of glacier foreland into fertile soils is enhanced by rhizodeposition, root exudation, decaying biomass, and root mass development. Safe-sites can be severely affected by geological dynamics, such as sudden mudslides, alluvial fan sliding, and scree movement, that take back the habitat to an earlier pioneer condition. Consequently, safe-sites cannot reach the climax but only a stable stage of middle maturity [5].

Furthermore, pioneer plants could select rhizosphere microbial communities able to promote plant growth thanks to the interactions in nutrient cycling and carbon sequestration [6]. Nevertheless, in a natural ecosystem it is difficult to assess the effect of vegetation on the rhizosphere bacterial communities, especially in high mountain environments characterized by variable environmental parameters (successional stage, pH, rainfall, moisture, mineral composition, sampling season, and slope) within a size-limited area typical of early and transitional successional stages. The impact of single plants on microbial communities in an alpine glacier forefield has previously been studied to highlight the relationship between the rhizosphere bacterial communities of pioneer plants or of the related bare soil and the chronosequence [7–10]. In an early chronosequential stage, the rhizosphere microbial community of* Poa alpina* L. was strongly influenced by the environmental conditions but, in transition and mature stage, plants could select a specific microbial community [9]. Along a similar chronosequence, the pioneer plant* Leucanthemopsis alpina* (L.) Heywood showed an opposing rhizosphere effect with a specific microbial community in the early successional stage only [7]. The study of the spatial extent of* Lc. alpina* on the microbial community and on the physical-chemical parameters in an early successional stage (5, 10 years) did not exhibit significant trends, supporting the conclusion of Tscherko et al. [9].

However, in a safe-site, the pioneer vegetation interrelated in floristic consortia often exhibited ground stems and root tangle with large nets. In this case, a safe-site could be equaled to a transitional or even a mature grassland for root tangle and plant community structure. The floristic community effect in such a habitat was observed in natural as well as in artificial experimental sites [11]. Osanai et al. [12] demonstrated that cooccurring plant species from native grassland selected their microbial communities. The effect was generally smaller than for species that generally do not cooccur naturally, such as those from agricultural crop systems [13], improved grassland systems, or fertilized grassland fields [14, 15]. Nunan et al. [16] found a weak influence of plant community or no effect of plant species on the structure and diversity of the root-colonizing bacterial community when comparing five cooccurring grass species from an upland grazed grassland in Scotland. Moreover, topography and other uncharacterized environmental factors seemed to be main drivers of the bacterial community composition.

On the other hand, studies about the effect of plant cover on microbial community in cold environments regarded different ecological niches and pointed out the higher significance of environmental parameters than the influence of the floristic consortia. In Antarctic environments along a latitudinal gradient, bacterial diversity of dense vegetation from different locations was comparable whereas bacterial diversity of “fell-field” vegetation decreased with increasing latitude [17, 18]. In permafrost meadow, steppe, or desert steppe, soil characteristics were driving factors of the microbial diversity [19]. In high elevation arid grassland, a strong plant effect was demonstrated for the perennial bunchgrasses* Stipa*,* Hilaria*, and for the invading annual grass* Bromus* [20].

Consequently, the aim of this work was to investigate if, in different safe-sites on a deglaciated terrain of the same chronosequential age, floristic consortia could select specific rhizobacterial communities.

## 2. Materials and Methods

### 2.1. Study Site and Soil Samples

The study site is located in the upstream subcatchment of Saldur river (46° 46′ 30′′ N; 10° 41′ 46′′ E; 2,400 a.s.l.) in the high Matsch valley (South Tyrol, Italy) with a drainage area of 11 km^2^. The main geological processes are periglacial and the streamflow is characterized by the glacier dynamics. During 1970–2000, the valley had an average rainfall of about 550 mm per year. In 2011, the mean temperatures of the plant growing season were 7.3°C in July, 10.3°C in August, and 8°C in September and the mean precipitations were 2.7, 2.5, and 3.6 mm per day, respectively. The dominant rock types are schist and gneiss [21] and the most common soil types are acidic leptosols, regosols, and umbrisols (mean pH = 4.3) derived from carbonate-free bedrocks. The study site, a foreland of about 3.3 Km^2^ left after a quick glacier retreat in the last 160 years [22], was located above the tree line (2,100 m a.s.l). The analysis of the historical maps of the third Austro-Hungarian topographic survey (the so-called “Franzisco-Josephinische Landesaufnahme”) dated 1850 and the aerial photographs of 1945 and of 2006 orthophotos were helpful to reconstruct the different stages of glacier retreat. Thus, comparing these photos, our sampling site was ice-free since 1850.

Rhizosphere and soil sampling were carried out in 2011 May, at the beginning of the plant growing season. Three safe-sites (RW, FI, and M sites) characterized by loosely organized assemblages of different plant species and a bare soil (BS site) were sampled. The sites were less than 20 × 20 cm. RW site, below an iron rich rock-face, was colonized by* Diphasiastrum alpinum* and* Gnaphalium supinum* L.; FI site, a floristic island between big rocks, was colonized by* Cladonia* sp.,* Festuca halleri* All.,* Polytrichum* sp.,* Racomitrium* sp.,* Sedum alpestre* Vill., and* Senecio carniolicus* (Willd.) Braun-Blanq.; M site, a safe-site surrounded by big rocks and characterized by a flatter area, was colonized by* Cetraria islandica* (L.) Ach.,* Leucanthemopsis alpina* (L.) Heywood,* Potentilla aurea* L.,* Rhododendron ferrugineum*,* Sibbaldia procumbens* L., and* Silene acaulis* (L.) Jacq. These sampling sites were carefully chosen in order to share similar conditions in terms of altitude, features, and geology.

The rhizosphere samples of all the single plant individuals within a floristic community were collected. Each individual plant was carefully pulled out the soil, without damaging its single root system. After pulling out each plant and avoiding roots, 4 g of rhizosphere soil strictly adhering to the roots was collected with a pair of sterile tweezers. Three replicates of bulk soil were collected as a control. Moreover, from each safe-site, 50 g of root-free soil was collected and put into plastic bags for soil chemical analysis. All the samples were immediately transported in refrigerated boxes to the laboratory as soon as the logistic constraints permitted and they were stored at −80°C until analysis.

### 2.2. Soil Chemical Analysis

Soil samples for chemical analysis were oven-dried at 105°C until constant weight and then acid was digested (HNO_3_ concentrated 65% and H_2_O_2_ 30%) in a milestone high performance microwave oven (MLS Mega, Gemini BV Laboratory, Apeldoorn, The Netherlands). To determine the total organic carbon content, soil samples were also acidified with HCl (6 M) to eliminate all carbonates. Metals and total phosphorous were determined by inductively coupled plasma-optical emission spectroscopy (ICP-OES, Spectro Ciros CCD, Spectro GmbH, Kleve, Germany). Nitrogen and C were quantified with an elemental analyzer (Flash 2000, Thermo Scientific). The pH_H_2_O_ was measured using an Accumet AP85 pH (Fisher Scientific Ltd., Pittsburgh, PA, USA). To test the level of significance of the observed chemical differences among sites, a Kruskal-Wallis test was done by using Mann-Whitney pairwise comparison post hoc test and Bonferroni correction in Past software [23].

### 2.3. Molecular Analysis of the Bacterial Communities

Total DNA of the rhizosphere and soil samples was extracted using Ultraclean Soil DNA Extraction kit (MO-BIO, Arcore, Italy). Microbial analyses were carried out using denaturing gradient gel electrophoresis (DGGE) [24] to describe the rhizobacterial taxa diversity and automated ribosomal intergenic spacer analysis (ARISA) [25] to describe the structure of the rhizobacterial communities.

For DGGE analysis, primers GC357f and 907r were used as described [26]. DGGE was run in a BioRad DCode universal mutation detection system (Bio-Rad, Milan, Italy). Polyacrylamide gels were done according to Muyzer et al. [24]. Gels were stained for 30 min in 1x TAE buffer containing SYBR Safe-DNA gel stain (Invitrogen, Milan, Italy). Visualization and digital image recording were performed with UVTec (Cambridge, UK). All the visible DGGE bands were excised and reamplified [24]. Sequencing was performed by STAB-Vida Inc. (Caparica, Portugal). Identification of 16S rRNA genes was done by comparison with EMBL/Genebank/DDBJ database and RDP database using BLASTN and Classifier, respectively. All sequences were submitted to the Ribosomal Database Project (RDP) web server [27] to assign taxonomy. Sequences were submitted to the Genbank/EMBL/DDBJ databanks under the accession numbers HG763876-HG764130.

ARISA fingerprint was performed as described by Cardinale et al. [25] with the ITSF/ITSREub primer set. Denatured ARISA fragments were run by STAB-Vida Inc. The data were analyzed with Peak Scanner software v1.0 (Applied Biosystems, Monza, Italy) and a threshold of 40 fluorescent units was used, corresponding to two times the highest peak detected during the negative control run. Output matrix was obtained as in Rees et al. [28].

### 2.4. Statistical Analysis of ARISA and DGGE

ARISA matrix was normalized with the formula (*x*/*∑x*)∗1000, where “*x*” is the fragment height in units of fluorescence, and then transformed on a logarithmic scale for multivariate analysis. Log-transformation was used to stabilize the sample variance, to reduce the interaction effect, and to normalize the distribution of data. Moreover, log-transformation can combine the information of a binary matrix with those of a nontransformed data matrix, hence preserving the relative abundance information and down-weighting dominant groups.

In order to assess changes in rhizobacterial community structure between floristic consortia, nonmetric multidimensional scaling (NMDS) was applied with Bray-Curtis algorithm. NMDS does not need the assumption of linear associations among variables being described as the most efficient ordination method for microbial ecology [29]. Bray-Curtis is not influenced by recurrent absent values into the matrix, a characteristic very common in ARISA matrices [30]. ANOSIM (based on Bray-Curtis similarity) was performed to test significant differences in the profile composition of the different sites. ANOSIM is a nonparametric statistical test, based on permutation, which uses rank similarity matrix of an ordination plot to calculate an *R* test statistic on the null hypothesis *H* ^0^ that there are no differences among groups. When *R* is near to 0, *H* ^0^ is true, while when *R* is reaching 1, *H* ^0^ can be rejected and there is a discrimination between groups. When ANOSIM statistics approaches 1, the similarities within groups are larger than the similarity between groups. We rejected *H* ^0^ when significance *P* value was <0.05. To test the level of significance between/within plant species ARISA clusters, a Kruskal-Wallis test was done as above.

The Nexus format of the phylogenetic tree of the DGGE identified bands performed by MEGA5 was submitted to the UniFrac web server to test differences among sites based in the UniFrac metric with 100 permutations and the Bonferroni correction factor [31]. A principal coordinates analysis (PCoA) on the DGGE sequence distance matrix for each pair of safe-sites was calculated through UniFrac metric. On the basis of the DGGE sequences, similar safe-sites tended to cluster together. In order to allow a broader view of those similarities, the first three principal components were considered.

## 3. Results

### 3.1. Soil Chemical Analysis

Soil resulted to be a sandy silt soil with an average texture of 72.3 ± 5.0% of sand, 21.0 ± 4.1% of silt, 6.6 ± 1.3% of clay, and 4.6 ± 1.3% of humus; pH was 4.5 ± 0.3%. Average chemical composition of sampled soils was total P 0.7 ± 0.1 mg/kg d.m., total K 7.4 ± 1.0 mg/kg d.m., total Ca 3.4 ± 0.6 mg/kg d.m., total Mg 13.4 ± 1.7 mg/kg d.m., total Fe 45.4 ± 6.9 mg/kg d.m., and total Al 29.4 ± 5.6 mg/kg d.m. No calcium carbonate was detected. Since those safe-sites were located in proximity of each other, their soil chemical composition did not differ substantially between sites (Kruskal-Wallis test *P* < 0.05; data not shown).

No nitrate was detected, while all the nitrogen found was represented by ammonia only. Nitrogen increased along an ideal gradient from bare soil (0.05% dry weight) to the most vegetated M site (0.98% dry weight) and also total organic carbon grew up from BS site (0.62% dry weight) to M site (19.3% dry weight; Table 1). The trend was confirmed by the C/N ratio which tended to increase constantly among sites of more complex vegetative patterns. Bonferroni-corrected Kruskal-Wallis nonparametric analysis of variance showed significant differences among sites for both total nitrogen, organic carbon content and C/N ratio, except for C and N content between RW versus FI and RW versus M (*P* values shown in Table 2) explained by a higher standard deviation of C and N content in M sites.

### 3.2. Genetic Structure of Bacterial Communities in Alpine Bulk Soils and Plant Colonized Safe-Sites

**Figure 1:**
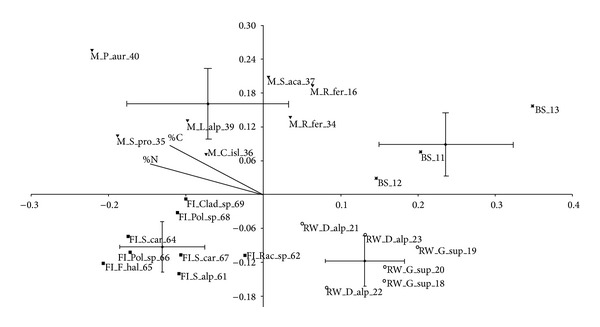
NMDS plot of the three safe-sites and the bare soil site according to UniFrac distance matrix. BS site was a root-free safe-site, RW site was an early developmental floristic stage, FI site was an intermediate stage, and M site was a later stage. Plant sample names are the following: C_isl—*Cetraria islandica* (L.) Ach.; Clad_sp—*Cladonia* sp.; D_alp—*Diphasiastrum alpinum*; F_hal—*Festuca halleri* All.; G-sup—*Gnaphalium supinum* L.; L_alp—*Leucanthemopsis alpina* (L.) Heywood; Pol_sp—*Polytrichum* sp.; P_aur—*Potentilla aurea* L.; Rac_sp—*Racomitrium* sp.; R_fer—*Rhododendron ferrugineum*; S_alp—*Sedum alpestre* Vill.; S_car—*Senecio carniolicus* (Willd.) Braun-Blanq.; S_pro—*Sibbaldia procumbens* L.; S_aca—*Silene acaulis* (L.) Jacq.

Due to the high sensitivity of the automated capillary electrophoresis, ARISA fingerprints of both rhizosphere and bare soil bacterial communities provided complex profiles with peaks ranging from 151 bp to 1437 bp and the 16S-23S rRNA internal transcribed spacer region (ITS) richness varied from 43 to 168 peaks. The electropherograms, characterized by distinct peaks number and intensity, revealed a shift in bacterial community structure across the different safe-sites plant communities. On the NMDS plot (stress value = 0.18), samples from root-free soil (BS), safe-site of early developmental stage (RW), intermediate stage (FI), and from the most mature one (M) showed four separate clusters based on microbial community structure (Figure 1). According to axis 1, RW site and BS site are separated from M and FI sites. According to axis 2, BS and M sites are separated by RW and FI sites. The unvegetated BS site clustered in a specific group, differentiated by the plant rhizospheres, is clustering closer to the rhizosphere bacterial communities of RW site than to those of FI and M sites. The NMDS separation is partially explained by N and C content, as shown by those variable vectors, which influenced more the M site than the other safe-sites. ANOSIM analysis confirmed a highly significant difference among the four microbial community structures (*R* = 0.81; *P* = 0.0001) and the performed test showed significant differences in the pairwise comparisons of the sites with *R* values approaching 1 in most of the cases (Table 3). Where replicated individuals of the same plant species within each safe-site were found, it was possible to denote a plant species effect. This is recognizable within RW safe-site, where individuals from* D. alpinum* and* G. supinum* formed two clusters significantly different along the first axis of NMDS (*P* = 0.032 at the Kruskal-Wallis test). In FI and M sites the tendency of individuals of the same species to cluster together seems to disappear, except for* R. ferrugineum*, maybe due to the higher number of species interconnected in the safe-site.

### 3.3. Diversity of the Bacterial Communities Associated with Alpine Bulk Soils and Pioneer Plants in Safe-Sites

**Figure 2:**
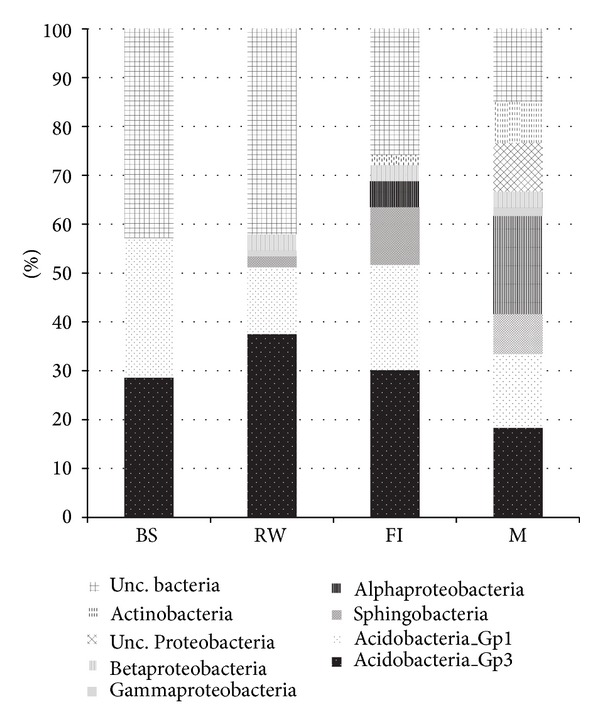
Percentage abundance of each taxonomic group for each individual rhizobacterial communities of the three safe-sites (RW, FI, and M) and the bare soil site (BS) after 16S rRNA gene DGGE-PCR analysis and band sequencing.

DGGE was performed to investigate the different microenvironments of the three safe-sites and bulk soil in terms of their dominant bacterial population composition. A total of 255 sequences of more than 300 bp were obtained from all sample profiles. RDP facilitated the determination of putative taxonomic affiliation of the recovered sequences. Major bacterial taxa included Acidobacteria* Gp3* and* Gp1*, Sphingobacteria, Alphaproteobacteria, Betaproteobacteria, Gammaproteobacteria, and Actinobacteria (Figure 2). A noteworthy amount of uncultured bacteria was found. Shifts in bacterial communities were visible. Members of the Acidobacteria order were present in all the sites samples. They generally represented the most abundant taxon, although a decrease of their relative abundance is visible with percentage from BS site (57%) to M site (33%). Proteobacteria were not found in BS site, while they were scarcely present in RW and FI site rhizospheres (4%, 8%, resp.). In M site Proteobacteria became more abundant than Acidobacteria (35%). In particular, the increasing abundance of* Proteobacteria* was due to Alphaproteobacteria, being more represented than Gammaproteobacteria and Betaproteobacteria. A considerable amount of unclassified Proteobacteria was also evident in M site. Sphingobacteria were recovered with low percentages in RW, FI, and M sites rhizospheres whereas members of Actinobacteria taxa were even less abundant being present in FI and M sites rhizospheres only. We did not find Sphingobacteria or Actinobacteria taxa associated with BS samples. According to RDP classification, unclassified Acidobacteria or Proteobacteria, as well as other uncharacterized bacteria, were quite common within all sites. For example, RW site was almost completely colonized by unclassified Acidobacteria and unknown Bacteria, except few sequences affiliated to uncultured* Burkholderia* or to a* Chitinophagaceae* bacterium. Similarly, FI safe-site was mostly colonized by unclassified Acidobacteria, although more frequent sequences belonging to Bradyrhizobiaceae,* Chitinophagaceae*, and other rarer taxa such as* Flavisolibacter* sp. or* Granulicella* sp. were found. Finally, M site, the most differentiated safe-site, counted the presence of unknown* Bradyrhizobiaceae*,* Bradyrhizobium* sp., and uncultured* Rhizobiales*, as well as* Chitinophagaceae*,* Streptacidiphilus* sp., Thermomonosporaceae, and Xanthomonadaceae.

**Figure 3:**
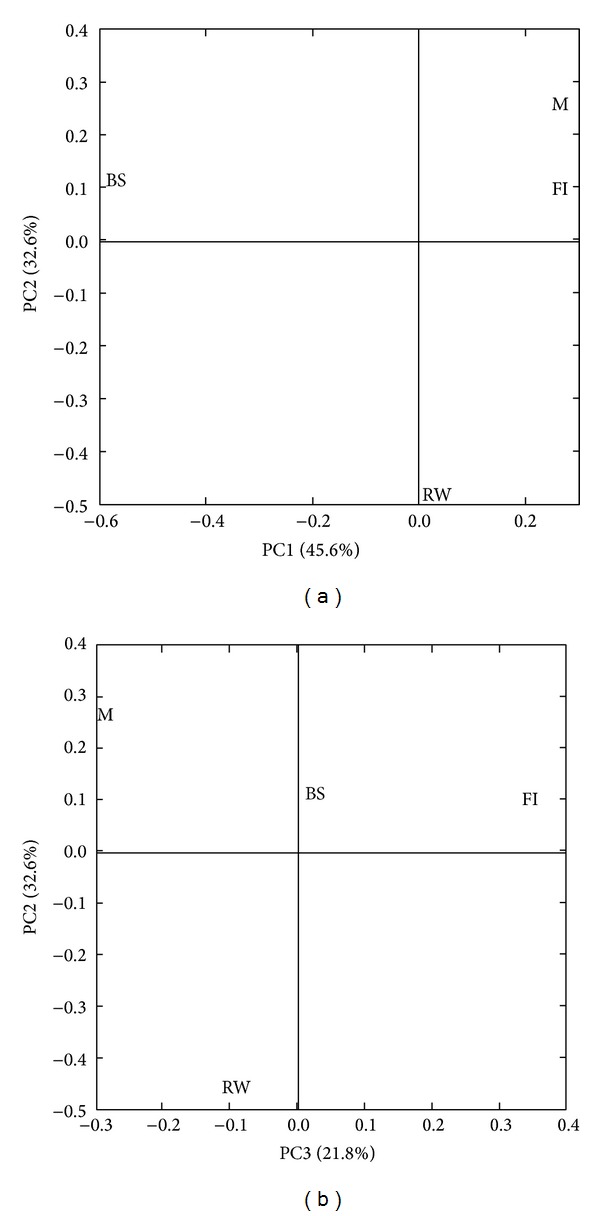
Principal coordinates analysis of the UniFrac distance matrix calculated to assess the overall sequence population similarity among safe-sites. Percentage of variance of the single principal coordinates axis is indicated.

Despite bias associated with sampling, DNA extraction, PCR amplification, and DGGE run, the pattern of differences in bacterial communities composition between the unvegetated soils of the BS site and the rhizospheres of the three safe-sites was supported by the pairwise UniFrac distance ordinations. Comparing each pair of environments using the Bonferroni correction, the UniFrac permutation test significance (*P* values < 0.05) showed that the BS site samples were significantly different from FI and M sites rhizospheres, but not from RW site rhizospheres. Moreover, the FI site rhizosphere did not differ significantly from M and RW sites rhizospheres, while the M site rhizospheres exhibited significant differences with the RW site. A PCoA analysis of the UniFrac distance matrix was calculated to assess the overall sequence population similarity among safe-sites (Figure 3). The first axis of PCoA analysis, explaining 45.6% of the total variance, showed a shift of the BS site from RW, FI, and M sites. The FI and M communities were located very close together in the same quadrant suggesting a similar bacterial community composition influenced by variables related to PC1. On the other hand, PC2 (32.6% of the variation) explained the differences between RW site and the other three sites. Finally, the third component (21.8% of the variance) differentiated FI from M and from BS and RW sites.

## 4. Discussion

Safe-sites are defined as environments immediately nearby a pool of seeds, where their germination, growth, and establishment are favorable [4]. In this respect, their availability, accessibility, and geomorphological diversity in high mountain represent important characteristics of this environment, since they represent a microsite where a list of ecological hazards (snow, wind, frost, and irradiation) are less severe than in open terrains and where plant propagules can resist, grow, and reproduce. In Matsch valley, belonging to south Tyrolean Alps, additional ecological hazards are represented by hot and dry summers, instability of the soil substrate, and excessive animal grazing [32]. Within each safe-site, more than one plant species can grow from seeds, specialized vegetative propagules, or plant fragments [33]. In such kind of environments, pioneer plants tend to grow in very complex coenosis, where roots are strictly intermixed and interrelated. A great diversity of root exudates from all these plants is released in rhizosphere, increasing the carbon amount of the safe-site. Due to the characteristics of safe-sites, usually well isolated among each other by rocks, sand, or mud, an analysis to understand the occurrence of a vegetation effect on rhizobacterial communities cannot be done with traditional squared-plots, where more safe-sites are sampled smoothing possible differences between them. Hence, we decided to study three kinds of safe-sites at different stages of morphological development, by sampling each single rhizosphere from all the growing plant individuals.

The vegetation complexity of the three safe-sites (RW, FI, and M) raised from a simple colonization of two species (RW site) to the colonization of lichens, mosses, and few herbaceous plant species (FI site) till M site, where five herbaceous species and one woody species (*R. ferrugineum*) were found. We discovered a distinct clustering of bacterial communities according to RW, FI, and M vegetation types that are significantly diverse from the unvegetated soil (BS site). We also found that a gradient in terms of C and N enrichments from BS site to the most developed M site was an important determinant of microbial community profiles. UniFrac analysis showed site-shifts in bacterial diversity which suggest a specialized physiology adapted to the peculiar site environmental conditions. Moreover, the differences among safe-sites, according to C and N gradients, support the occurrence of a plant cover effect on the rhizosphere bacterial community within those safe-sites.

Previous investigations of the rhizosphere effect were conducted on few single pioneer plants or in grassland plots. Almost all the researches on the rhizosphere effect associated with a single plant species were achieved on crop or other plants either in artificial microcosms such as pots or on agricultural soils such as orchards and crop monocultures. Most of these researches demonstrated that peculiar root exudation and rhizodeposition of different plant species could select the structural and functional diversity of the associated rhizosphere bacterial communities [34–36]. On the other hand, a consistent number of studies have showed that several environmental parameters, that is, soil type, soil characteristics, growth stage, management practices, and growing season may influence the composition of the microbial communities in the rhizosphere [37–44]. Past studies about a natural alpine ecosystem investigated single plant species along successional chronosequences and found inconsistent effects of pioneer plants on rhizosphere microbial communities. For example, while the rhizobacterial community of* Lc. alpina* was different from the interspace community in an early successional chronosequential stage, in a later stage it became similar to the interspace community. In this case, it seemed that the influence of* Lc. alpina* depended on soil age and that nutrient availability could influence the bacterial community structure [7]. In another study case,* Lc. alpina* individuals in the early successional stage (5, 10 years) of a glacier forefield showed no selective effect on the microbial community, since a similar bacterial community structure was apparent up to 40 cm of distance to the plant [8]. Another single pioneer plant,* P. alpina*, did not exhibit a selective role on its rhizosphere bacterial community in the pioneer stage of a chronosequence, maybe due to the harsh environmental conditions of the plot where it was growing [9]. However, by investigating a more mature soil, the same plant species could select a specific microbial community but related to soil properties and carbon supply.

On the other hand, safe-sites are more complex than single pioneer plant individuals in a cold environment, but they show less complexity than a homogeneous plot carefully designed in mountain grasslands. Real safe-sites are much less homogeneous, being shaped by the history of the microarea where they are such as dynamical differences in climate, in geophysical features, or in biota colonization which determine complicated patterns and often unique rates of soil development [1]. In our case, due to the quick glacier melting in the last 80 years, the 160-year soil represents the only transitional step of the glacier moraine between earliest stages (<10 years) and mature soil (>500 years). As shown by aerial photos, orthophotos, and a topographic survey, one of the glacier tongues of the Weisskugel glacier has been retreating with a discontinuous movement. Consequently, there was no constant gradient of soil age but distinct block stages where soil age is invariable. In this sense, the 160-year-old stage is more stable than an earlier successional soil and it can host a larger number of plant species. Nevertheless it was possible to distinguish hundreds of safe-sites of which the three chosen were the most represented. Within the stable block stage of 160 years old, the measured differences in rhizobacterial composition and soil parameters supported the hypothesis that the plant community composition of each floristic consortium exhibited an effect on the rhizobacterial communities widely documented in studies done in quite different ecosystems. For example, Nunan et al. [16] demonstrated a more important influence of the plant community composition than of the individual plant species on the root colonizing bacterial community in an upland grazed grassland, whereas Osanai et al. [12] showed a significant impact of the plant species on the soil bacterial community composition. Similar results were obtained comparing the rhizosphere bacterial communities of three plant species of an arid grassland [20].

The rhizosphere bacterial communities of RW site, characterized by only two different plant species, clustered more closely with the BS site than with the vegetated ones showing a simpler bacterial community, as confirmed by the UniFrac analysis which detected no significant difference between the two sites. Although FI and M sites had a similarity of about 56%, inside the FI site were found rhizobacterial communities of mosses and lichens which did not cluster strictly with the plant ones. The presence of lichens and mosses in the same site could explain why the bacterial community of the FI site represented an intermediate stage between the RW site and the M site. The M site, colonized by individuals of six plant species, could be considered a later stage where floristic consortia selected a more complex bacterial community which significantly differs from the one of BS and RW sites. The UniFrac analysis showed that the BS communities were distinct from ones of the FI and M sites and were weakly similar to the ones of RW site. Moreover, the intermediate plant colonization stage, FI site, did not differ significantly from the RW and the M vegetated sites. Previous studies [9, 45, 46] showed that the development of the soil microbial community in alpine glaciers was determined by the accumulation of soil TOC and total nitrogen. The increasing content of C and N in the floristic consortia corresponded with increased floristic developmental stage. Soil nutrients and C influenced the bacterial community composition along a chronosequence [7], while in the Mendenhall glacier chronosequence [47] they were not correlated with the rhizobacterial communities. These different conclusions seem to strongly depend on the adopted experimental design. Cultural-independent techniques based on phospholipid fatty acid (PLFA) determination [9, 10], to point out the different concentration of bacterial/fungal fatty acids and to compare the Gram-positives/Gram-negatives ratio, or molecular methods like restriction fragment length polymorphism (RFLP) and DGGE analyses [7, 8] could not have enough resolution to detect little changes in the bacterial community genetic structure due to faint environmental variables [48]. The ARISA analysis we used, targeting the intergenic 16S-23S rRNA gene highly variable ITS region, showed more sensitivity and enabled the detection up to subspecies level, increasing the chance of the analysis to detect very little effects on complex bacterial communities [49].

## 5. Conclusions

Despite the harsh environmental condition of the natural alpine ecosystem and the tight complex root system of the safe-site, our results support the capability of different pioneer plant consortia to select specific rhizobacterial communities with an increase of bacterial diversity according to the increase of soil maturation. Moreover, when plants of the same species occurred in the same site, the associated rhizobacterial communities clustered more strictly together according to their genetic structures, confirming the high similarity of the rhizobacterial communities within individuals of the same pioneer plant species.

## Figures and Tables

**Table 1:** Percentage of total nitrogen and carbon content and C/N ratio in the four safe-sites.

Safe-site	Nitrogen %		Carbon %		C/N	
	Average	St. dev.	Average	St. dev.	Average	St. dev.
BS	0.05	0.01	0.62	0.16	11.5	0.61
RW	0.27	0.11	3.48	1.47	12.7	0.94
FI	0.72	0.35	10.4	6.03	14.2	1.46
M	0.98	0.85	19.3	18.3	17.5	3.79

**Table 2:** Level of significance (*P* values) of the differences in C, N, and C/N content among sites by Bonferroni-corrected Kruskal-Wallis test.

	C	N	C/N
BS versus RW	0.023	0.023	0.023
BS versus FI	0.028	0.028	0.043
BS versus M	0.019	0.019	0.032
RW versus FI	0.175	0.197	0.012
RW versus M	0.772	0.954	0.023
FI versus M	0.004	0.004	0.045

**Table 3:** *P* and *R* values of ANOSIM based on Bray-Curtis similarity of the four safe-sites as grouped after ARISA-NMDS plot analysis.

*P*/*R* value	BS	RW	FI	M
BS		0.9630	0.9758	0.7937
RW	0.0124		0.9390	0.7434
FI	0.0077	0.0005		0.7055
M	0.0092	0.0009	0.0004	
